# Muscle Force Contributions to Anterior Cruciate Ligament Loading

**DOI:** 10.1007/s40279-022-01674-3

**Published:** 2022-04-18

**Authors:** Nirav Maniar, Michael H. Cole, Adam L. Bryant, David A. Opar

**Affiliations:** 1 School of Behavioural and Health Sciences, Australian Catholic University, 17 Young Street, Fitzroy, VIC 3065 Australia; 2 Sports Performance, Recovery, Injury and New Technologies (SPRINT) Research Centre, Australian Catholic University, Melbourne, Australia; 3 School of Behavioural and Health Sciences, Australian Catholic University, Brisbane, Australia; 4 Healthy Brain and Mind Research Centre, Australian Catholic University, Melbourne, Australia; 5 Department of Physiotherapy, Centre for Health, Exercise and Sports Medicine, The University of Melbourne, Melbourne, Australia

## Abstract

Anterior cruciate ligament (ACL) injuries are one of the most common knee pathologies sustained during athletic participation and are characterised by long convalescence periods and associated financial burden. Muscles have the ability to increase or decrease the mechanical loads on the ACL, and thus are viable targets for preventative interventions. However, the relationship between muscle forces and ACL loading has been investigated by many different studies, often with differing methods and conclusions. Subsequently, this review aimed to summarise the evidence of the relationship between muscle force and ACL loading. A range of studies were found that investigated muscle and ACL loading during controlled knee flexion, as well as a range of weightbearing tasks such as walking, lunging, sidestep cutting, landing and jumping. The quadriceps and the gastrocnemius were found to increase load on the ACL by inducing anterior shear forces at the tibia, particularly when the knee is extended. The hamstrings and soleus appeared to unload the ACL by generating posterior tibial shear force; however, for the hamstrings, this effect was contingent on the knee being flexed greater than ~ 20° to 30°. The gluteus medius was consistently shown to oppose the knee valgus moment (thus unloading the ACL) to a magnitude greater than any other muscle. Very little evidence was found for other muscle groups with respect to their contribution to the loading or unloading of the ACL. It is recommended that interventions aiming to reduce the risk of ACL injury consider specifically targeting the function of the hamstrings, soleus and gluteus medius.

**Supplementary Information:**

The online version contains supplementary material available at 10.1007/s40279-022-01674-3.

## Key Points

**Table:** 

The hamstrings and soleus are effective at unloading the ACL by generating posterior shear forces at the tibia. For the hamstrings, this effect is contingent on knee flexion angle exceeding 20° to 30°.
The gluteus medius opposes the knee valgus moment more than any other muscle during weightbearing tasks, thus unloading the ACL.
The quadriceps and gastrocnemius tend to increase load on the ACL by inducing anterior shear forces at the tibia. For the quadriceps, this effect only exists when the knee is flexed less than ~ 50°.
The majority of the evidence comes from cadaveric and musculoskeletal modelling studies, with less evidence from in vivo methods.

## Introduction

Anterior cruciate ligament (ACL) injuries are one of the most common knee pathologies sustained during athletic participation [1], with incidence rates reported to be 0.05 and 0.08 per 1000 exposures for males and females, respectively [2]. These injuries are particularly prevalent in sports that require frequent changes of direction and landing tasks, such as basketball, soccer, football and hockey [2] and are associated with substantial convalescence [3] and costs [4, 5]. Thus, development of effective strategies for ACL injury prevention is vital [6].

ACL rupture occurs when the mechanical load experienced by the ligament exceeds the ligament’s ability to withstand that mechanical load. The mechanisms by which the ACL experiences mechanical load have therefore been of primary interest in video analysis of injury scenarios [7–13], biomechanical and simulation studies [14–16], and cadaveric studies [17–20]. These studies have identified key knee joint loading parameters (e.g., anterior shear forces) that have been associated with markers of ACL loading (e.g., ACL force or strain); however, direct strategies to mitigate these mechanical loads have remained elusive.

Whilst ACL loads are known to vary passively (i.e., as a function of the knee flexion angle), muscles produce forces that can modulate (i.e., accentuate and/or reduce) mechanical loads at the knee, and therefore play a critical role in dictating the size and nature of the loads experienced by the ACL. Therefore, effective training of specific muscles may mitigate ACL loads during catastrophic injurious scenarios, thus protecting the ACL from injury. ACL rupture may also occur as a consequence of repetitive cyclic loading leading to microdamage (fatigue failure) rather than a single catastrophic occurrence [21], and thus knowledge of how to modulate ACL loads during non-injurious scenarios may also directly inform ACL injury preventative interventions. An important first step for such interventions would be to understand how individual muscles contribute to ACL loading. Therefore, the purpose of this narrative review was to summarise the existing evidence to determine how specific lower limb muscles contribute to ACL loads. The specific aims of this review are to:

1. Provide a brief primer on knee joint biomechanics;
2. Describe the injury and loading mechanisms of the ACL;
3. Provide an overview of methodological considerations for studies that investigate the roles of muscle force and ACL loading;
4. Provide an overview of the current literature pertaining to the relationship between lower limb muscle force and ACL loading.

## Knee Joint Biomechanics

**Fig. 1:**
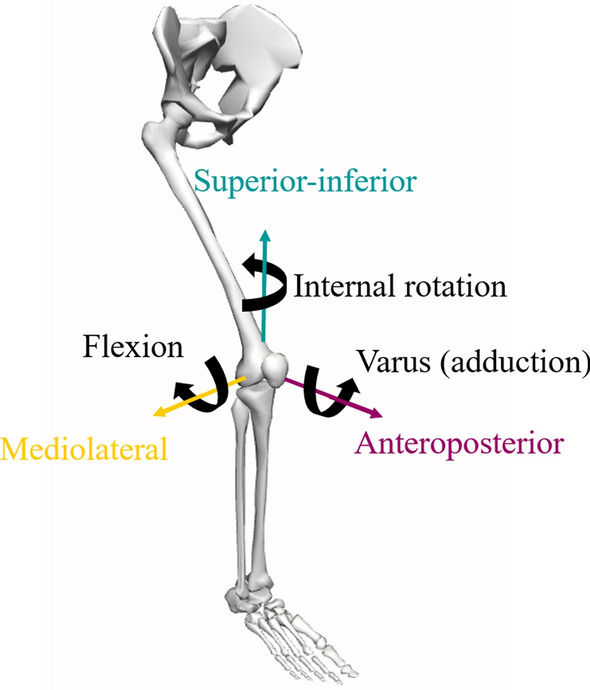
Illustration of knee joint degrees of freedom using a previously described musculoskeletal model [30] in OpenSim [31]

Although motion at the knee can occur within six degrees of freedom, these can be described about three principal axes in which the tibia may translate along or rotate about (Fig. 1). The anteroposterior axis allows anteroposterior translation and valgus-varus rotation. The vertical axis allows superior-inferior translation and internal–external rotation. The mediolateral axis allows mediolateral translation and knee flexion and extension. Range of motion for each of these movements is limited by various structural factors associated with the knee joint, including the bone shape and various soft-tissue structures (i.e., ligament, cartilage and muscle/tendon). During weightbearing tasks (e.g., walking and landing), healthy knees exhibit relatively small translational knee range of motion (< 6 mm), whilst rotational motion is highly plane specific [22]. Valgus-varus and internal–external rotation typically display small excursions of up to 5° and 20°, respectively [22]. In the sagittal plane, knee flexion angles typically range from 0° (full extension) to 90° for the weightbearing leg in most locomotive tasks [22–27], although excursions exceeding this do also occur in commonly performed tasks such as the swing phase of stair ambulation [27] and high-speed running [28]. Although the primary role of the ACL is to resist anterior tibial translation [29], prior investigations of ACL injury and loading mechanisms (see Sect. 3) suggest that other movements across all three axes, as well as the forces and moments that cause them, should be considered. For the present review, we describe forces and moments along and about these axes based on the movements that they induce on the tibia. For example, a valgus moment induces valgus rotation of the tibia relative to the femur, and an anterior shear force induces anterior translation of the tibia relative to the femur.

## Injury and Loading Mechanisms of the Anterior Cruciate Ligament (ACL)

Research analysing sports-related video/television footage of ACL injuries has found the common mechanism of ACL injury to be non-contact dynamic tasks, such as single-leg landings, sudden decelerations and rapid change-of-direction manoeuvres [7–13]. In particular, ACL injury tends to occur shortly after initial contact [8], where the knee joint experiences relatively large degrees of knee valgus and rotation (either internal or external) and high mechanical loads [8–13]. Although video-based observations have limited accuracy [32], these findings are consistent with cadaveric studies showing that frontal and transverse plane knee mechanics influence ACL loading [17, 33–35].

In the frontal plane, higher knee valgus or varus moments both have the potential to increase loads on the ACL [33, 35]. However, knee valgus collapse has been reported to be the more common mechanism of injury in video-based analyses [7, 8, 13]. In the transverse plane, an internal rotation moment of the tibia with respect to the femur has been found to expose the ACL to higher loads than an external rotation moment [33, 35]. Moreover, non-sagittal plane knee joint moments have been shown to have the greatest influence on ACL loading when they occur simultaneously, and especially in conjunction with an anterior shear force [17, 33, 35, 36]. As the primary role of the ACL is to resist anterior translation of the tibia relative to the femur [29], it is unsurprising that anterior and posterior shear forces have been consistently shown to load and unload the ACL, respectively [17, 33, 34, 37–40]. Knee joint compression is also thought to play a role in ACL injury. Early work suggested that increases in joint compression would be favourable due to decreased tibial translation [41]; however, more recent work has shown that compression may increase strain on the ACL, due to the posterior slope of the tibia [42–44].

As a consequence of this research, anterior translation, knee valgus and knee internal rotation (or the forces and moments that produce these) are often considered surrogate markers for ACL loading. Therefore, understanding how muscles contribute to or oppose these knee joint loads may provide insight into how these muscles load and unload the ACL.

## Methodological Considerations

To assess how muscle force may contribute to ACL loading, studies have used in vitro, in silico and in vivo methods. For this review, in vitro experiments refer to studies involving cadavers (sometimes also referred to as in situ), in silico experiments refer to studies that involve simulation techniques, and in vivo experiments refer to studies involving living organisms. Each of these methods is associated with distinct advantages and limitations. Hence, prior to summarising the relevant findings, the methods used in these studies must first be scrutinised. Note that a summary of the studies included in this review is provided in Supplementary Tables 1–3 (see electronic supplementary material [ESM]).

### In vitro

Numerous studies have adopted an in vitro approach to investigate the relationship between muscle force and ACL loading [34, 45–63]. Via robotic manipulation of cadaveric knees, these studies can alter joint angles of cadaveric knees to simulate a variety of knee angles [48, 49, 54, 55, 63] and realistic movements such as high-impact landings [46, 47]. Muscle forces can be simulated via cables or springs attached at the site of the muscle of interest. However, these cables often apply static forces [46, 47, 49, 54, 55] that are not representative of in vivo conditions. Additionally, since only the knee is evaluated and/or manipulated, these methods do not account for full-body kinematics and typically represent only knee-spanning muscles. This particular limitation is notable, as non-knee-spanning muscles can contribute to knee joint loads via a phenomenon known as dynamic coupling, whereby a force acting on any one point on the body is transmitted to other segments in the body via joint intersegmental forces [64]. However, unlike in vivo approaches, in vitro investigations can load tissues to the point of failure [20]; thus, actual injury thresholds can be determined as long as the cadaveric tissues are appropriately preserved. Nevertheless, data pertaining to ACL injury thresholds and failure loads should be interpreted with consideration of the characteristics of the cadavers, as tissue properties differ with aspects such as age [65] and sex [66].

### In silico

In silico approaches use computer simulation techniques that often rely on data collection (e.g., via motion capture data) from healthy organisms. In the context of muscle forces and ACL loading, this involves employing musculoskeletal modelling [26, 67–79] and/or finite element modelling [71, 80–84] techniques. The use of these techniques offers several distinct advantages. Firstly, modelling enables the investigation of cause–effect research questions that are otherwise impractical or impossible to directly assess [31]. For example, using an in silico approach, it is possible to assess the relationship between muscle force and joint loading during dynamic tasks such as walking [73, 85, 86]. Additionally, musculoskeletal modelling can overcome some limitations of cadaveric approaches, whereby interactions between muscle forces and whole-body skeletal dynamics can be accounted for [64]. As such, the contribution of both knee-spanning and non-knee-spanning muscles to knee joint loading can be assessed by determining muscular contributions to ground reaction forces (GRF) [26, 73, 75, 86], thereby accounting for dynamic coupling. However, validation of these musculoskeletal simulations poses a fundamental challenge for the research community, as this method is generally based on numerous assumptions and uncertainties [87]. For example, muscle forces can be estimated via a variety of electromyography (EMG)-driven [88] and optimisation [89–91] approaches. However, given muscle forces cannot practically be measured in vivo, direct validation of muscle forces is not possible. Nevertheless, best practice recommendations for validation and verification of musculoskeletal simulations are available [87]. For example, indirect validation of muscle forces is possible via comparison of the estimated and experimentally measured joint contact forces (e.g., in participants with instrumented joint implants), due to the high dependency of joint contact loads on muscle forces [92]. Subsequently, modelling studies should be interpreted following careful consideration of the validation and verification procedures.

### In vivo

In vivo assessments of muscle force contributions to ACL loading can be conducted via invasive and non-invasive protocols; however, the methodological approach used by these studies is often unique to each investigation. ACL strain can be calculated directly via surgical placement of a differential variable reluctance transducer on the ACL fibres, with electrical stimulation of muscles to assess the relationship between muscle force and ACL strain [93]. The primary advantage of this approach is direct measurement of ACL strain in vivo, along with controlled stimulation of muscles. However, this method is highly invasive and participants require general anaesthesia. Moreover, only sub-injurious muscle forces can be assessed, and not during clinically relevant high-demand sporting manoeuvres (e.g., sidestep cutting and single-leg landing). The way in which a muscle contributes to joint loading is dependent on the orientation of all segments in the system [64]; thus, failing to account for whole-body kinematics during high-demand sporting manoeuvres limits the application of the findings from these methods.

Non-invasive in vivo methods are possible, although they require indirect methods for quantifying ACL strain and/or muscle force contribution. A series of studies [94–97] have used surface or, more invasively, intramuscular EMG to indirectly assess the contribution of particular muscles to ACL loading. Serpell and colleagues [96] coupled surface EMG analysis with fluoroscopy to assess tibiofemoral skeletal movement during a single-leg step-up and tracked ACL attachment sites in order to estimate ACL strain. Other studies incorporated frontal plane knee moments derived via a dynamometer [94, 95] or inverse dynamics [97] as a surrogate marker of ACL loading during isometric action and assessed associations with EMG activity of knee-spanning muscles. The major limitation of these studies was that the muscle force was not controlled and, clearly, none of the experiments performed their assessments during high-demand sporting manoeuvres. However, EMG data can be processed to obtain in silico estimates of muscle force [72, 88], and the highly accurate fluoroscopy technique [98, 99] has been used to track tibiofemoral kinematics during drop landings [100]. These methods therefore warrant further research. One study [61] used a KT-1000 arthrometer to monitor anterior tibial translation in response to different passive muscle forces from the ankle plantar-flexors. These muscle forces, however, were not directly monitored since force was altered indirectly by changing the ankle dorsi-flexion angle. Whilst this highlights a common limitation of in vivo experiments (i.e., limited ability to control independent variables), the strength of in vivo work lies in its ability to represent the organism in its native environment. Whilst in vitro and in silico approaches attempt to do this, their ability to achieve this is limited by their inherent limitations and assumptions.

## Search strategy

A retrospective, citation-based methodology [101] was used to obtain articles from databases such as PubMed and Google Scholar. Search terms included those related to the ACL (‘anterior cruciate ligament’, ‘ACL’, ‘knee’, ‘tibiofemoral’) as well as muscle, ligament or joint loading (‘strain’, ‘force’, ‘shear’, ‘translation’, ‘rotation’, ‘valgus’, ‘abduction’, ‘muscle force’, ‘muscle contributions’, ‘muscle induced’). Only peer-reviewed literature in English were considered. In this review, we only included articles that specifically determined the role of lower limb muscle forces on ACL force/strain, or any other of the previously described surrogate markers of ACL loading, in humans. No specific assessment of methodological quality was performed.

## The Role of Muscles in ACL Loading

Despite the aforementioned limitations of in vitro, in silico and in vivo methods, it is noted that many of the limitations inherent with each method are often complemented by the strengths of alternate methods. As such, should relatively consistent findings be reported using different techniques across studies, this would provide some confidence in the validity of the reported outcomes. The following section provides an overview of findings from studies using various methodologies in an effort to describe the role of different lower limb muscles in ACL loading.

### Quadriceps

**Fig. 2:**
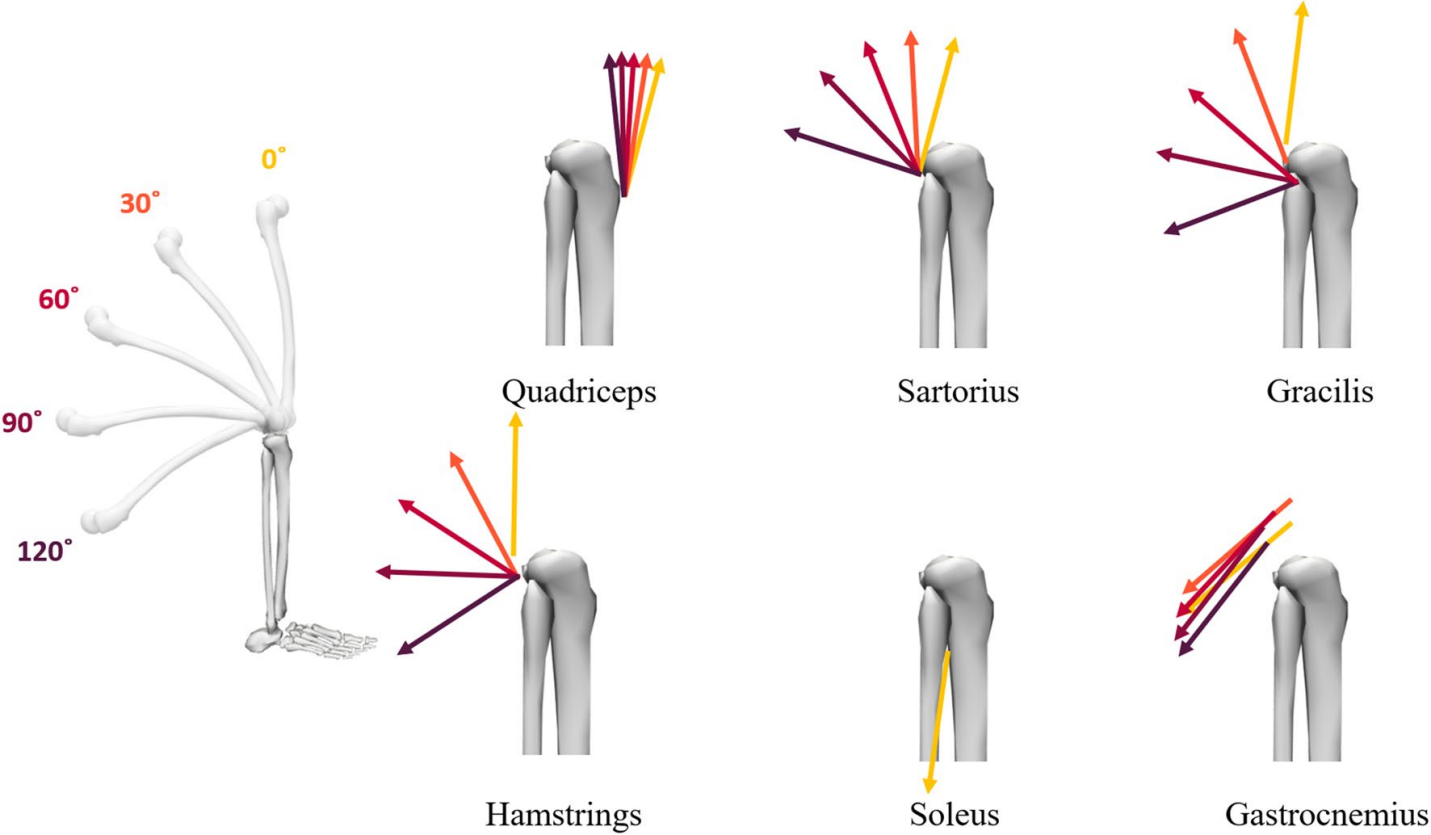
Illustration of force unit vectors acting at the tibiofemoral joint of knee-spanning muscles from knee flexion angle of 0° (full extension) to 120° of flexion. Unit vectors were derived from a previously published and validated musculoskeletal model [30] using a previously described tool [107] in OpenSim [31]. Note that unit vectors are visualised at their effective attachment points (as opposed to their anatomical attachment points), which accounts for wrapping surfaces and can therefore change as a function of the knee flexion angle. For muscles with multiple actuator components (quadriceps, hamstrings and gastrocnemius), the average attachment point and unit force vector was visualised. As the soleus did not span the knee, its unit vector was invariable due to the changing knee flexion angle. All illustrated unit vectors represent attachments to the shank, with the exception of the gastrocnemius which attaches to the femur

The quadriceps are one of the most extensively investigated muscle groups in relation to ACL loading [46–50, 53–56, 59, 63, 69, 70, 73, 74, 80, 81, 102] and studies have consistently shown that the force produced by this muscle group significantly contributes to the loads on the ACL. For example, Withrow and colleagues [46] showed that in vitro ACL strain during simulated impact landings had a strong positive correlation (*R*^2^ = 0.74) with the change in quadriceps tendon force. However, the influence of quadriceps force appears to be dependent on the knee flexion angle (see Fig. 2 for illustration). At lower knee flexion angles (i.e., less than ~ 30° to 50°), in vitro and in silico evidence shows that quadriceps force induces ACL loading [49, 53, 55, 57–59, 69, 81], anterior shear force [74], anterior tibial translation [54, 56, 60, 62, 63], knee valgus rotation [56, 60], knee valgus moment [73, 74] and tibial internal rotation [54, 56, 60, 62, 63]. This collectively suggests that the quadriceps tend to be an antagonist to the ACL. However, at very high knee flexion angles (i.e., greater than ~ 80°), the quadriceps have a limited role in ACL loading and may even serve to unload this structure [49, 53, 59, 69] due to the changing angle between the patella tendon and the longitudinal axis of the tibia at increased knee flexion angles [62, 63, 70] (Fig. 2). During potentially injurious manoeuvres such as sidestep cutting and single-leg landing, the observed knee flexion angles are typically < 70° in the weightbearing leg, and are therefore compatible with an anteriorly directed quadriceps force vector relative to the tibia. Such tasks also require large quadriceps muscle forces [23, 67], collectively suggesting that the quadriceps are likely to produce an anterior shear force, and thus ACL loading, during these tasks. Musculoskeletal modelling studies support this, demonstrating that the quadriceps typically induce anteriorly directed shear forces of up to 233 N and 342 N (more than any other muscle group) during sidestep cutting [75] and single-leg landing [26], respectively. Similarly, the quadriceps have also been observed to be the greatest contributor to the anterior shear force in simulation studies of a drop-lateral jump (~ 1070 N at the time of peak ACL force) [103] and bilateral drop landing (up to ~ 577 N) [104], although neither of these studies appeared to consider shear force contributions for any non-knee-spanning muscles. Peel and colleagues [76] used a musculoskeletal modelling approach to support these findings in a stop-jump task, further exploring individual quadriceps muscles and showing that the vastus lateralis produced the greatest contribution to ACL force (0.89 bodyweights of force) compared with the other quadriceps (≤ 0.17 bodyweights each). During a bilateral drop jump, Ueno and colleagues [82] used a finite element model to show greater peak ACL strain and force when quadriceps muscle force was simulated (ACL strain = 7.2%, ACL force = 479 N), compared with identical conditions (i.e., same kinematics and GRFs) but with all muscles (ACL strain = 3.3%, ACL force = 195 N) or just hamstring muscle force (ACL strain = 2.6%, ACL force = 171 N) simulated.

Three musculoskeletal modelling studies appear to support these aforementioned findings but have notable limitations. During a weightbearing forward lunge, a musculoskeletal modelling study showed that the quadriceps generate more anterior shear force than any other muscle [79]. However, the musculoskeletal model employed was characterised by a patella tendon angle (relative to the longitudinal axis of the tibia) that was greater than that observed in cadaveric [105] or in vivo [106] experiments. Subsequently, the anterior shear force contributions of the quadriceps were likely overestimated. Two studies employed modelling approaches that explored ‘high risk’ and ‘low risk’ (as defined by the peak ACL force or strain) simulations of ski landing [78] or a bilateral drop jump [84]. During the ski jump [78], the ‘high risk’ condition had higher vasti activation, but also had lower hamstring and soleus activation. During the drop-jump simulations [84], the ‘high risk’ condition had lower muscle forces, with the exception of the vastus intermedius (as well as the iliopsoas and medial gastrocnemius). Whilst both of these studies suggest an association between quadriceps activation/force and ACL loading, the simultaneous changes in the activation/force of other muscles likely confound the conclusions. Additionally, both of these studies allowed joint kinematics to vary between conditions, meaning the observed changes in ACL loading in these studies cannot be attributed solely to muscle force changes.

### Hamstrings

The hamstrings have received substantial attention in the literature given their potential to unload the ACL [47–52, 54, 55, 58, 67–70, 80, 96, 102, 108, 109]. This is primarily due to the hamstrings’ ability to produce a posterior shear force at the tibia [48, 74, 109]. Hence, the majority of studies have investigated the role of hamstring-quadriceps co-contraction to assess whether the hamstrings can reduce the injurious loads imposed by the quadriceps muscle group [47, 48, 52, 54, 55, 58, 63, 70, 96, 108]. These studies (both in vitro and in silico) have consistently reported that hamstring co-contraction can have a protective effect by reducing ACL strains and forces [47, 55, 58, 80, 102], anterior shear forces [48, 108, 109], anterior tibial translation [48, 52, 54, 63] and internal tibial rotation [48, 52, 54, 63].

Like the quadriceps, the effectiveness of hamstring contraction at influencing ACL loading is dependent on the knee flexion angle (Fig. 2). Near full extension, the hamstrings are relatively ineffective at producing a posterior shear force due to their line of action and small mechanical advantage in this position [102]. At knee flexion angles of greater than ~ 20° to 30°, the hamstrings are more effective at producing posterior shear forces, thus unloading the ACL [48, 49, 52–55, 102]. The poor mechanical advantage of the hamstrings (at producing posterior shear force) at low knee flexion angles, coupled with the strong mechanical advantage of the quadriceps in this position (for generating anterior shear force), may partly explain observations of ACL injury promptly after initial contact, where knee flexion angles are typically low [8]. However, computational modelling studies have demonstrated that hamstring muscle force induces substantial posterior shear force during the weight acceptance phase of sidestep cutting (up to 188 N, knee flexion angle = 21°–42°) [75], the landing phase of single-leg landing (up to 469 N, knee flexion angle = 15°–70°) [26, 67], during the landing phase of a bilateral drop landing (up to ~ 693 N, knee flexion angle = 34°–93°) [104] and at the time of peak estimated ACL force during a drop-lateral jump (~ 430 N, knee flexion angle =  ~ 42°) [103]. Importantly, these posterior shear force contributions were greater than any other lower limb muscle [26, 75], but two of these studies only considered knee-spanning muscles [103, 104]. The previously described finite element modelling study [82] (see Sect. 6.1) showed that the addition of hamstring force produced lower peak ACL loading (ACL strain = 2.6%, ACL force = 171 N, occurring at knee flexion angles of less than ~ 73°) during a bilateral drop jump, when compared with simulations without any muscle forces (ACL strain = 6.8%, ACL force = 418 N) or with only quadriceps forces (ACL strain = 7.2%, ACL force = 479 N). Additional in silico work has shown that reduced hamstring strength (as a result of a fatiguing protocol) was associated with increased estimated peak ACL forces (821 N) compared with the pre-fatigue condition (605 N) during the first 100 ms of foot contact during sidestep cutting (knee flexion angles of ~ 30°), but changes in kinematics (e.g., hip flexion angles) and kinetics (e.g., GRFs) between conditions may confound these results [68]. In contrast, one musculoskeletal modelling study [76] found that hamstring muscle forces contribute to ACL force during a stop jump task. This counter-intuitive finding could represent task-specific differences in hamstring function but may also be explained by methodological differences. Collectively, this suggests that the hamstrings are capable of reducing ACL loading at critical timepoints during some potentially injurious manoeuvres. Despite this, it is important to recognise that each of these simulation studies were based on motion analysis data collected from healthy participants that safely completed these manoeuvres. During actual injury scenarios, baleful movement mechanics may limit the hamstrings’ protective capacity to induce posterior shear force at the tibia.

**Fig. 3:**
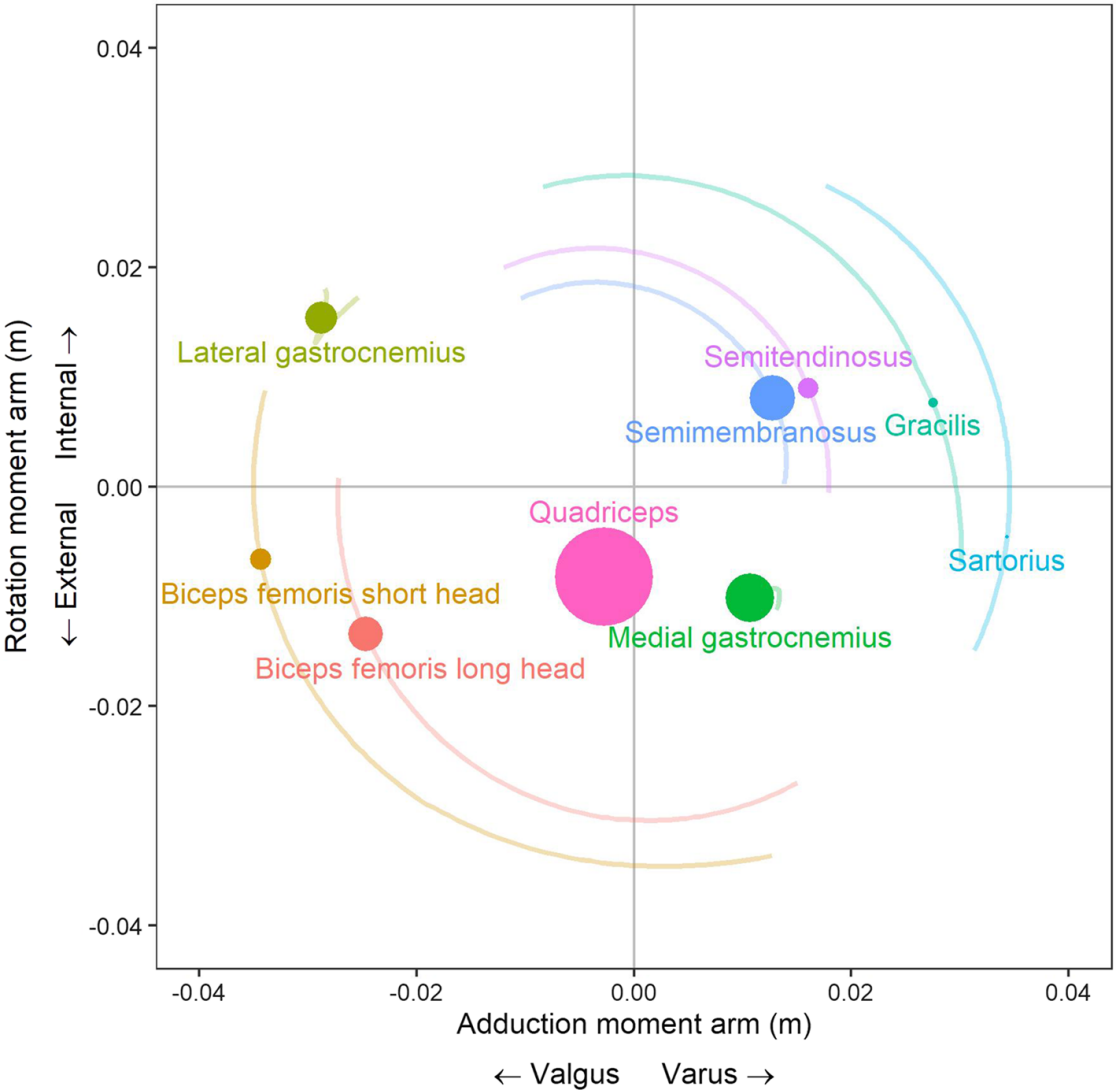
Frontal (*x*-axis) and transverse (*y*-axis) plane tibiofemoral moment arms of knee-spanning muscles derived from a previously validated musculoskeletal model [30]. Circles indicate the moment arm of each muscle at 30° of knee flexion; circle size is proportional to the muscle’s corresponding physiological cross-sectional area derived from Ward et al. [111]; faded lines show the change in moment arm across the knee flexion range (0°–120°)

In the frontal and transverse planes, each hamstring muscle has a potentially different influence on ACL loading, owing to their different attachment sites and moment arms relative to the knee joint [110] (Fig. 3). In the frontal plane, the medial (semimembranosus and semitendinosus) and lateral (biceps femoris) hamstrings appear to exert varus and valgus loading at the knee, respectively, during both sidestep cutting [75] and single-leg landing [26]. In vivo work using EMG data supports this, showing that hamstring and quadriceps co-contraction may play a role in limiting externally applied valgus and varus loading at the knee [72]. However, these roles appear limited in magnitude relative to other muscles, such as the gluteus medius, which produces peak knee varus moments that are 6.4- and 2.3-fold greater than the medial hamstrings during sidestep cutting [75] and landing [26], respectively.

One simulation [109] and one in vitro study [51] have both shown that the biceps femoris group has the greatest ability to unload the ACL given its ability to oppose internal rotation of the knee [51], its large capacity (relative to the semitendinosus) to generate muscle force [109] and ability to produce adequately sized posterior shear forces [51, 109]. Compared with the biceps femoris, the orientation of the semimembranosus limits its ability to oppose ACL loading, whilst the semitendinosus is heavily limited by its relatively small physiological cross-sectional area [109–111]. Whilst this suggests greater relative importance of the biceps femoris, an in vivo approach [96] provides contrasting conclusions during a step-up task, finding that higher medial hamstring to quadriceps EMG activation patterns were associated with lower ACL strain (as determined from bi-planar fluoroscopy), whilst the opposite was true for the lateral hamstring to quadriceps EMG activation ratio. This particular finding is limited since muscle force was not directly quantified or controlled in this study, and additional research is needed to further elucidate the role of the individual hamstring muscles and their relative importance for unloading the ACL. Additionally, the relatively small physiological cross-sectional area of the semitendinosus is not unchangeable, and considerably larger cross-sectional areas in this muscle have been observed in trained athletes [112]. This suggests that modelling studies may benefit from greater participant specificity.

### Gastrocnemius

The role of the gastrocnemius in ACL loading is somewhat contentious [45, 50, 61, 67, 71, 74, 93–95, 97, 102]. An in vivo approach [93] showed that direct electrical stimulation of the gastrocnemius induced greater ACL strain at knee flexion angles of 15°, 30° and 45° relative to the ACL strain induced by a relaxed gastrocnemius. Whilst this study did not investigate knee flexion angles of > 45° due to experimental limitations, visualisation of the gastrocnemius force vector at the femur (Fig. 3) suggests that this effect would likely persist throughout the knee flexion angle range. Both simulation [71] and in vitro [45] studies support this, showing that gastrocnemius muscle force acts as an ACL antagonist across the majority of the knee flexion range by inducing increased ACL force and anterior tibial translation, respectively. Moreover, simulation studies have also shown that the gastrocnemius acts as an ACL antagonist by inducing an anterior shear force on the tibia (or equivalently, inducing anterior tibial translation) during single-leg landings [26, 67], unanticipated sidestep cutting [75], bilateral drop jumps [113], bilateral drop landings [104], drop-lateral jumps [103] and walking [74, 83]. Importantly, the induced anterior shear force from the gastrocnemius is of comparable magnitude (up to 334 N) to that of the quadriceps group (up to 342 N) during single-leg landing [26] and drop-lateral jumps (~ 1065 N vs ~ 1070 N) [103], but less so for sidestep cutting (117 N vs 233 N) [75] and bilateral drop landing (~ 144 N vs ~ 577 N) [104]. During a bilateral drop jump task, the gastrocnemius was found to have the greatest contribution to peak anterior shear forces in one musculoskeletal modelling study [113], but another [76] reported ‘negligible’ contributions to ACL forces during a stop-jump task when compared with other muscles like the quadriceps. During walking, one modelling study suggested that a relatively greater gastrocnemius force condition (5.3 N·kg^−1^ vs 2.8 N·kg^−1^) was associated with higher anterior tibial translation (3.9 mm vs 3.0 mm) and ACL force (3.4 N·kg^−1^ vs 2.4 N·kg^−1^) at 50% of the stance phase [83]. However, the modelling approach employed in this study allowed other muscles forces to simultaneously vary, meaning the observed changes in ACL loading may be attributed to observed changes in quadriceps, hamstring and soleus muscle force. Collectively, this suggests that the task may influence the relative importance of the gastrocnemius and the quadriceps muscle groups, respectively, for generating anterior shear forces and/or ACL forces. Despite these relatively consistent findings, contrary findings have been observed. Durselen and colleagues [50] used an in vitro approach to determine that gastrocnemius force did not contribute to ACL strain at knee flexion angles of 0°–110°. However, these findings may be explained by the low applied muscle forces (gastrocnemius forces = ~ 40 to 50 N). Whilst these forces are much lower than what would be expected in vivo, the authors reported that low muscle forces were needed to avoid cadaveric tissue failure. Teng and colleagues [114] used electrical stimulation to observe the effects of gastrocnemius force on ultrasound-measured anterior tibial translation and found no significant differences between four different gastrocnemius stimulation intensities (ranging from the lowest intensity necessary to elicit a twitch to the maximum tolerable intensity). A musculoskeletal modelling study [115] of single-leg landings concluded that the gastrocnemius may protect the ACL owing to higher observed gastrocnemius muscle forces in ‘low risk’ compared with ‘high risk’ trials (peak ACL force = 1152 N vs 2092 N); however the mean difference in peak gastrocnemius muscle force was of a very low magnitude (52 N) and not statistically significant.

In the frontal and transverse planes, EMG data suggest that the medial gastrocnemius tends to induce knee varus and external rotation loading (thus oppose knee valgus and internal rotation loading), whilst the lateral gastrocnemius plays the opposite role [94, 95, 97] owing to their opposing moment arms (Fig. 3). Simulation studies tend to support these potential stabilising roles in dynamic movements such as walking [73, 74], sidestep cutting [75] and single-leg landing [26]. These contributions, however, are relatively insubstantial compared with other muscles such as the gluteus medius (described in Sect. 6.5), which produces peak knee varus moments that are 13.2- and 2.6-fold greater than the medial gastrocnemius during sidestep cutting [75] and single-leg landing [26], respectively.

### Soleus

Despite not crossing the knee joint, a growing body of literature suggests that the soleus can meaningfully unload the ACL. An in vivo study [61] found that passively dorsi-flexing the ankle resulted in reduced anterior tibial translation measured using a KT-1000 arthrometer, concluding that the ankle plantar-flexors play a role in stabilising the knee against anterior tibial translation (as dorsi-flexing the ankle produces passive force in the ankle plantar-flexors). The authors replicated this test in cadavers in the same study [61] and found that the influence of ankle dorsi-flexion on tibial translation persisted when the gastrocnemius was cut, but not when the soleus was cut, suggesting the observed effect was more likely due to the soleus [61]. Four other studies have also suggested that the soleus may help to unload the ACL. In an in vitro study [45], investigators dissected the soleus from the gastrocnemius in cadavers and demonstrated that soleus muscle force caused posterior translation of the tibia. Using a computational musculoskeletal model, two studies [26, 67] have shown that the soleus produces a posterior shear force at the tibia during single-leg drop landing. A similar modelling approach supports these findings during unanticipated sidestep cutting [75]. Whilst the contributions of the soleus to posterior shear may be perceived to be small (due to the line of action of the soleus relative to the tibia; Fig. 2), the soleus’ large physiological cross-sectional area and primary anatomical function (i.e., to produce ankle plantar flexion) mean that it produces considerable muscle force magnitudes during dynamic weightbearing tasks [23, 67]. Consequently, the soleus makes substantial contributions to the GRF [23], and thus (via dynamic coupling) makes sizeable contributions to the posterior shear force at the tibia. Computational simulations suggest that these contributions are up to 173 N and 393 N during sidestep cutting [75] and single-leg landing [26], respectively, making their posterior shear force contributions second only to the hamstrings in both of these tasks. In contrast, one recent modelling study [76] found that the soleus contributes to ACL forces during a stop-jump task. This study also found similarly counterintuitive results for the hamstrings (see Sect. 6.2), suggesting that methodological differences are the most likely explanation for the dissimilar findings compared with other literature.

In the frontal and transverse plane, the role of soleus force on ACL loading is less clear. In vivo and in vitro data investigating these relationships are lacking, whilst in silico data provide conflicting evidence. For example, soleus muscle forces have been shown to induce knee valgus loading during the late stance phase of walking [73] and the weight acceptance phase of sidestep cutting [75]. However, another study showed the soleus induced knee varus loading during single-leg landing [26]. Similarly, the role of the soleus in the transverse plane has been found to be different in sidestep cutting [75] and single-leg landing [26]. Although these opposing findings may represent task-based specificity in the frontal and transverse plane roles of the soleus, these differences could also be attributable to differences in modelling practices (e.g., different foot–ground contact models). Subsequently, although the evidence tends to suggest that the soleus has the potential to unload the ACL in the sagittal plane, further work is needed to elucidate its role in the frontal and transverse planes. In particular, in vivo and in vitro approaches may be needed to overcome limitations associated with in silico methods, which may be sensitive to modelling assumptions.

### Gluteal Muscle Group

The gluteal muscle group, especially the gluteus medius, has been identified as an important contributor to resisting dynamic knee valgus collapse, via its role as a hip abductor [116]. This hypothesis is somewhat supported by a prospective study [117] that demonstrated increased risk of ACL injury in association with lower hip abduction strength (a known action of the gluteal muscle group). However, investigation of gluteal muscle force and its association with knee joint loading is limited to in silico investigations. A recent modelling study found that a greater knee valgus moment was associated with lower gluteus medius muscle force (as part of a multivariate regression model) during a drop vertical jump [118]. This same research group utilised a finite element modelling approach that showed ‘high risk’ (i.e., higher ACL strain) bilateral drop jump simulations were associated with lower gluteus medius force [84]; however, the ‘high risk’ condition was also characterised by potentially confounding differences in kinematics and kinetics compared with the ‘low risk’ condition. Another modelling study [77] found that hip abductor fatigue (and thus, lower simulated gluteus medius force) resulted in no changes in estimated ACL force during single-leg landing compared with baseline trials, but did observe a significantly greater external knee valgus and knee external rotation moment. However, compensatory increases in muscle forces (e.g., increased semitendinosus and vastus intermedius force) and other observed biomechanical differences (e.g., changes in trunk kinematics) under the fatigued condition may have confounded these results. Additionally, three simulation studies [26, 73, 75] have directly investigated contributions of the gluteal muscle group to knee loading during dynamic tasks. During walking, it was found that the gluteus medius was the dominant contributor to the knee varus moment (thus opposing knee valgus loading) via its contribution to the GRF [73]. The same role for the gluteus medius was subsequently described in more potentially injurious manoeuvres, with varus contributions of up to 32 Nm during the weight acceptance phase of unanticipated sidestep cutting [75] and 38 Nm during the landing phase of a single-leg drop landing task [26]. Most importantly, this role of the gluteus medius was 8- and 2.5-fold greater than any knee-spanning muscle investigated in sidestep cutting [75] and single-leg landing [26], respectively. These studies also show similar findings for the other muscles in the gluteal region (e.g., gluteus maximus, gluteus minimus and piriformis), but their relative importance is inconsistent across studies [26, 73, 75]. Like other non-knee-spanning muscles, the notable absence of in vivo and in vitro research may limit the strength of these findings. However, the consistent findings across studies suggest that the role of the gluteus medius in opposing knee valgus loading is relatively robust.

In the transverse plane and sagittal plane, the role of the gluteal muscles in modulating ACL loading appears to be less substantial in both sidestep cutting and single-leg landing [26, 75]. For example, the gluteal muscles appear to make small (< 10 Nm) contributions to the knee internal rotation moment [26, 75]. Shear force contributions also appear to be small (< 24 N on average) for most of the gluteal group [26, 75]; however, the gluteus maximus tends to produce an anteriorly directed shear force in sidestep cutting (up to 55 N) and single-leg landing (up to 72 N). A study by Alkjaer and colleagues [79] provides contrasting evidence, showing that the gluteus maximus may play a substantial role in generating posterior shear forces at the tibia (during a lunge task) via its attachment to the iliotibial band (ITB). Most musculoskeletal modelling studies do not account for the gluteus maximus’ attachment to the ITB, which likely explains the contrasting findings. Subsequently, the role of the gluteus maximus in generating shear forces appears sensitive to the modelling of its attachment to the ITB. To better elucidate its role, further work that draws upon in vivo or in vitro methodologies may be needed to overcome the limitations associated with modelling assumptions.

### Other Muscles

Limited research suggests other knee-spanning muscles such as the tensor fasciae latae (via its attachment to the iliotibial band), sartorius and gracilis also influence ACL loading. Specifically, an EMG-based in vivo study conducted on a dynamometer suggested that the tensor fascia latae and sartorius muscles may induce knee valgus loading, whilst the gracilis tends to oppose this [94, 95]. However, these studies were based on isometric contractions, which may limit their applicability to dynamic injury mechanisms. Figure 2 also demonstrates that the gracilis and sartorius muscles induce posterior shear at the tibia for knee flexion angles greater than ~ 30°. However, these muscles have a relatively small physiological cross-sectional area [111, 119] (Fig. 3), suggesting that muscle force production, and therefore their induced reaction forces and moments at the knee, is likely limited. Musculoskeletal simulation studies support this notion, showing that these muscles produce negligible contributions to knee shear forces (< ~ 40 N) [26, 75, 103], frontal plane moments (< 4 Nm) [26, 75] or transverse plane moments (< 1 Nm) [26, 75] during dynamic tasks.

Similarly, other non-knee-spanning muscles appear to have limited roles in ACL loading when compared with the quadriceps, hamstrings, gastrocnemius, soleus and gluteal muscle groups. Musculoskeletal modelling studies have shown that the adductors (adductor brevis, longus and magnus) and ankle dorsi-flexors (tibialis anterior, extensor digitorum longus and extensor hallucis longus) contribute to anterior shear forces during single-leg landing (up to 72 N and 74 N, respectively) [26] and sidestep cutting (up to 58 N and 63 N, respectively) [75]. These same muscles produce a varus moment of up to 10–13 Nm during single-leg landing and sidestep cutting. The plantar-flexor invertors (tibialis posterior, flexor digitorum longus and flexor hallucis longus), iliopsoas and peroneus groups have also been investigated, but produce relatively small contributions to shear forces (≤ 14–36 N) and frontal plane moments (≤ 4–6 Nm) each during these same tasks. Contributions to tibial rotational moments for all of these non-knee-spanning muscles are insubstantial (≤ 4 Nm) during single-leg landing and sidestep cutting.

## Conclusion

Both knee-spanning and non-knee-spanning muscles can increase or decrease load on the ACL. In particular, the hamstrings, soleus and gluteus medius appear to have the greatest ability to oppose key markers of ACL loading, whilst the quadriceps and gastrocnemius appear to have the greatest ability to induce ACL loading (or surrogate markers of it). The hamstrings’ ability to generate posterior shear force at the tibia (thus unloading the ACL) exceeds all other muscles during weightbearing movement, but appears to be less effective when knee flexion angles are less than ~ 20° to 30°. This effect is notable since low knee flexion angles are commonly observed at the time of ACL injury, and also provide the quadriceps with their greatest mechanical advantage for generating anterior shear forces (thus increasing load on the ACL). The soleus’ role in generating posterior shear forces at the tibia, although secondary to the hamstrings, does not appear to be sensitive to the knee flexion angle and thus may represent an important agonist of the ACL during injury mechanisms associated with low knee flexion angles. Practically, however, the function of the soleus may be difficult to isolate from the gastrocnemius, which induces anterior shear forces at the tibia of considerable magnitudes during weightbearing movement (second only to the quadriceps). The gluteus medius makes small contributions to posterior shear forces, but its ability to oppose knee valgus loading (a key marker of ACL loading) has been consistently shown to exceed any other muscle during weightbearing movement. It is suggested that interventions aiming to mitigate risk of ACL injury consider targeting the function of these specific muscles.

## Supplementary Information

Below is the link to the electronic supplementary material.

Supplementary file1 (PDF 166 KB)

## Declarations

### Availability of data and material

All extracted data are available from the corresponding author upon reasonable request.
