# Supplementary material for: Muscle Force Contributions to Anterior Cruciate Ligament Loading
Source: Sports Med. 2022 Apr 18;52(8):1737–50. doi: 10.1007/s40279-022-01674-3 (PMC9325827; doi:10.1007/s40279-022-01674-3)
Supplement: Supplementary file 1 — Supplementary file1 (PDF 166 KB) [file 40279_2022_1674_MOESM1_ESM.pdf]

Supplementary material

This document contains supplementary material for the following document:

Title

Muscle force contributions to anterior cruciate ligament loading

Authors

Nirav Maniar[1,2], Michael H Cole[3,4], Adam L Bryant[5], David A Opar[1,2]

[1]School of Behavioural and Health Sciences, Australian Catholic University, Melbourne, Australia

[2]Sports Performance, Recovery, Injury and New Technologies (SPRINT) Research Centre, Australian Catholic University, Melbourne, Australia

[3]School of Behavioural and Health Sciences, Australian Catholic University, Brisbane, Australia

[4]Healthy Brain and Mind Research Centre, Australian Catholic University, Melbourne, Australia

[5] Centre for Health, Exercise and Sports Medicine, Department of Physiotherapy, The University of Melbourne, Melbourne, Australia

Corresponding author

Nirav Maniar

Nirav.Maniar@acu.edu.au

+61 3 9953 3030

17 Young Street

Fitzroy, VIC, Australia

3065

This document contains supplementary Tables 1-3, which cover methodological details for in-vitro (Supplementary Table S1), in-silico (Supplementary Table S2) and in-vivo (Supplementary Table S3) studies included in this review. Note that reference numbers within the tables correspond to the reference list in the main manuscript, for ease of cross-referencing. The reference list in this document only includes references that are not included in the main manuscript.

Supplementary Table 1. Summary of in-vitro studies discussed in review.

Study	n	Sex (% male)	Age	Task	Knee flexion (˚)	Weight bearing	Muscle force application	Marker of ACL load
Arms et al. (1984) [57]	17	76	54	Knee flexion	0 to 90	No	Cables	ACL strain
DeMorat et al. (2004) [56]	13	46	(49 to 93)	Static	20	No	Cables	ACL rupture, ATT, valgus°, tibial rotation°
Draganich et al. (1990) [58]	5	NR	65 ± 21	Knee flexion	0 to 90	No	Cables	ACL strain
Durselen et al. (1995) [50]	9	NR	(22 to 55)	Knee flexion	0 to 110	No	Cables	ACL strain
Elias et al. (2003) [45]	6	NR	(67 to 77)	Knee flexion	20 to 80	No*	Cables	ACL strain
Hirokawa et al. (1992) [62]	12	NR	NR	Knee flexion	0 to 120	No	Cables	ATT, tibial rotation°

Hsich et al. (1997) [60]	15	60	52 ± 15	Knee flexion	0 to 120	No*	Cables	ACL length, ATT, valgus°, tibial rotation°
Li et al. (1999) [54]	10	NR	(42 to 72)	Knee flexion	0 to 120	No	Cables	ACL force, ATT, tibial rotation°
Li et al. (2004) [55]	18	NR	(52 to 72)	Knee flexion	0 to 150	No	Cables	ACL force
MacWilliams et al. (1999) [48]	8	62	(58 to 91)	Knee flexion	15 to 70	Yes	Cables	ATT, tibial rotation°, shear force
Markolf et al. (1990) [34]	17	NR	(56 to 68)	Knee flexion	-5 to 45	No	Cables	ACL force
Markolf et al. (2004) [49]	13	77	(29 to 67)	Knee flexion	-5 to 120	No	Cables	ACL force
More et al. (1993) [52]	10	NR	70	Squat	0 to 90	Yes	Cables	ACL force, ATT, tibial rotation°

Renstrom et al. (1986) [53]	7	NR	(37 to 81)	Knee flexion	0 to 120	No	Cables	ACL strain
Sherbondy et al. (2003) [61]	4	NR	(70 to 78)	Dorsi-flexion	30	No	Passive forces	ATT
Victor et al. (2010) [63]	6	50	(78 to 87)	Knee flexion	30 to 120	No	Cables	ATT, valgus moment, internal rotation moment
Withrow et al. (2006) [46]	11	45	71 ± 19	Single-leg landing	25 to 31	Yes	Cables	ACL strain
Withrow et al. (2008) [47]	10	40	60 ± 24	Single-leg landing	25 to 31	Yes	Cables	ACL strain, ATT, valgus°, tibial rotation°

Abbreviations: ACL, anterior cruciate ligament; ATT, anterior tibial translation; NA, not applicable; NR, not reported.

Age data is presented as mean, mean ± standard deviation or the range (minimum to maximum) depending on data availability.

Knee flexion range is defined as a range (minimum to maximum), where 0˚ indicates full knee extension, positive values indicate flexion, negative value indicate hyperextension.

*Weight bearing was simulated but with insufficient magnitude to reflect realistic bodyweights; °Joint rotation angle in degrees.

Supplementary Table 2. Summary of in-silico studies discussed in review.

Study	n	Sex (% male)	Age	Task	Knee flexion (˚)	Weight bearing	Muscle force application	Marker of ACL load
Adouni et al. (2016) [71]	NA	NR	NA	Knee flexion	0 to 90	No	Simulated	ACL force
Adouni et al. (2016) [71]	NA	NR	NA	Gait	~0 to 5[#]	Yes	Simulated	ACL force
Alkjaer et al. (2012) [79]	1	0	20	Forward lunge	~40 to 116	Yes	Simulated	Shear force
Biscarini et al. (2013) [109]	NA	NR	NA	Leg extension exercise	0 to 90	No	Simulated	Shear force
Biscarini et al. (2014) [108]	20	100	(21 to 47)	Leg extension exercise	0 to 90	No	Simulated	Shear force
Heinrich et al. (2020) [78]	1	NR	NR	Bilateral ski-landing	~43 (at peak ACL) ~36 to 132	Yes	Simulated	ACL force

Kim et al. (2021) [77]	10	50	27 ± 1	Single-leg landing	~13 to 66	Yes	Simulated	ACL force, valgus moment, internal rotation moment
Maniar et al. (2018) [75]	8	100	27 ± 4	Sidestep cutting	21 to 42	Yes	Simulated	Shear force, valgus moment, internal rotation moment
Maniar et al. (2020) [26]	8	100	27 ± 4	Single-leg landing	15 to 70	Yes	Simulated	Shear force, valgus moment, internal rotation moment
Mesfar et al. (2005) [81]	NA	NR	NA	Knee flexion	0 to 90	No	Simulated	ACL force, ATT
Mesfar et al. (2006) [80]	NA	NR	NA	Knee flexion	0 to 90	No	Simulated	ACL force, ATT
Mokhtarzadeh et al. (2013) [67]	8	100	23 ± 1	Single-leg landing	23 to 65	Yes	Simulated	ACL force

Morgan et al. (2014) [115]	6	100	21 ± 2	Single-leg landing	14 to 61	Yes	Simulated	ACL force
Nasseri et al. (2021) [103]	24	0	20 ± 4	Drop-lateral jump	9 to 66	Yes	Simulated	Shear force
Navacchia et al., 2019 [113]	13	0	NR	Drop jump	~21 to 70^	Yes	Simulated	Shear force
O'Connor (1993) [102]	NA	NR	NA	Knee flexion	0 to 140	No	Simulated	ACL force
Pandy et al. (1997) [69]	NA	NR	NA	Knee flexion	0 to 90	No	Simulated	ACL force
Peel et al., (2021) [76]	10	0	24 ± 3	Stop-jump	~4 to 75	Yes	Simulated	ACL force
Pflum et al. (2004) [104]	1	100	28	Bilateral drop landing	~34-93	Yes	Simulated	Shear force
Sharifi & Shirazi-Adl (2021) [83]	NA	NR	NA	Gait	(50% stance) (75% stance)	Yes	Simulated	ACL force, anterior tibial translation
Shelburne et al. (1998) [70]	5	100	26 ± 3	Squat	0 to 90	Yes	Simulated	ACL force, shear force

Shelburne et al. (2005) [74]	5	100	26 ± 3	Gait	~3 to 35#	Yes	Simulated	ACL force, shear force
Sritharan et al. (2012) [73]	8	100	26 ± 4	Gait	~3 to 35#	Yes	Simulated	Valgus moment
Ueno et al., (2020) [118]	13	0	16 ± 2	Drop jump	~21 to 70^	Yes	Simulated	Valgus moment
Ueno et al., (2021) [82]	13	0	16 ± 2	Drop jump	~21 to 70^	Yes	Simulated	ACL strain, ACL force
Ueno et al., (2021) [84]	13	0	16 ± 2	Drop jump	~21 to 70^	Yes	Simulated	ACL strain,

Abbreviations: ACL, anterior cruciate ligament; ATT, anterior tibial translation; NA, not applicable; NR, not reported.

Age data is presented as mean, mean ± standard deviation or the range (minimum to maximum) depending on data availability. NA is given for studies that used modelling approaches where a single model was used.

Knee flexion range is defined as a range (minimum to maximum), where 0° indicates full knee extension, positive values indicate flexion, negative value indicate hyperextension. In some cases, knee flexion angles were not reported by the study, and were therefore estimated based on similar literature as follows: #estimated based on normative gait data [24]; ^Estimated from normative drop jump data [120].

Simulated muscle force application involves estimation via optimisation techniques, or directly specified by investigators.

Supplementary Table 3. Summary of in-vivo studies discussed in review.

Study	n	Sex (% male)	Age	Task	Knee flexion (˚)	Weight bearing	Muscle force application	Marker of ACL load
Flaxman et al. (2017) [97]	25	48	27 ± 7[c]	Force-direction	23	Yes	In-vivo (voluntary)	Valgus moment, internal rotation moment
Fleming et al. (2001) [93]	6	67	(38 to 56)	Knee flexion	5 to 45	No	In-vivo (stimulation)	ACL strain
Howe et al. (1990) [59]	5	100	(18 to 40)	Knee flexion	30 to 90	No	In-vivo (voluntary)	ACL strain
Lloyd et al. (2001) [95]	10	100	29 ± 5	Force-direction	40 to 90	No	In-vivo (voluntary)	Valgus moment, internal rotation moment
Lloyd et al. (2005) [72]	11	100	21 ± 3	Sidestep cutting	27 to 47	Yes	In-vivo (voluntary)	Valgus moment

Serpell et al. (2015) [96]	5	100	24 ± 4	Single-leg step up	NR	Yes	In-vivo (voluntary)	ACL length, ATT, valgusº, tibial rotationº
Sherbondy et al. (2003) [61]	12	58	(18 to 43)	Dorsi-flexion	30	No	In-vivo (passive)	ATT
Teng et al. (2021) [114]	10	100	23 ± 4	Prone-lying	10	No	In-vivo (stimulation)	ATT

Abbreviations: ACL, anterior cruciate ligament; ATT, anterior tibial translation; NA, not applicable; NR, not reported.

Age data is presented as mean, mean ± standard deviation or the range (minimum to maximum) depending on data availability. For one study [97], male and female data were reported separately, and were therefore pooled using the formula from the Cochrane handbook [121].

Knee flexion range is defined as a range (minimum to maximum), where 0˚ indicates full knee extension, positive values indicate flexion, negative value indicate hyperextension.

ºJoint rotation angle in degrees.

In-vivo muscle force were generated as either voluntary, voluntary contraction; passive, passive muscle forces; stimulation, contraction produced via electrical stimulation.

References

120. Ford KR, Myer GD, Schmitt LC, Uhl TL, Hewett TE. Preferential quadriceps activation in female athletes with incremental increases in landing intensity. J Appl Biomech. 2011;27(3):215-22.

121. The Cochrane Collaboration. Cochrane Handbook for Systematic Reviews of Interventions Version 5.1.0 [updated March 2011]. www.handbook.cochrane.org. 2011.
